# Mass spectrometry assays of plasma biomarkers to predict radiographic progression of knee osteoarthritis

**DOI:** 10.1186/s13075-014-0456-6

**Published:** 2014-10-07

**Authors:** Susan Y Ritter, Jamie Collins, Bryan Krastins, David Sarracino, Mary Lopez, Elena Losina, Antonios O Aliprantis

**Affiliations:** Department of Medicine, Division of Rheumatology, Immunology and Allergy, Brigham and Women’s Hospital, 75 Francis St, Boston, MA 02115 USA; Harvard Medical School, Longwood Ave, Boston, MA 02115 USA; Department of Orthopedic Surgery, Brigham and Women’s Hospital, 75 Francis St, Boston, MA 02115 USA; Thermo Fisher Scientific - BRIMS Center, Cambridge, MA 02139 USA

## Abstract

**Introduction:**

Biomarkers to identify osteoarthritis (OA) patients at risk for disease progression are needed. As part of a proteomic analysis of knee synovial fluid from normal and OA patients, differentially expressed proteins were identified that could represent potential biomarkers for OA. This study aimed to use mass spectrometry assays to identify representative peptides from several proteins in synovial fluid and peripheral blood, and assess their levels as biomarkers of OA progression.

**Methods:**

Multiplexed high throughput selected reaction monitoring (SRM) assays were developed to measure tryptic peptides representative of 23 proteins in matched serum and synovial fluid samples from late OA subjects at the time of joint replacement. Subsequently plasma samples from the baseline visit of 173 subjects in an observational OA cohort were tested by SRM for peptides from nine of these proteins: afamin, clusterin, cartilage oligomeric matrix protein, hepatocyte growth factor, kallistatin, insulin-like growth factor binding protein, acid labile subunit, lubricin, lumican, and pigment epithelium-derived factor. Linear regression was used to determine the association between the peptide biomarker level at baseline and change in joint space width (ΔJSW) from baseline to 30 months, adjusting for age and sex.

**Results:**

In the matched cohort, 17 proteins could be identified in synovial fluid and 16 proteins were detected in serum. For the progression cohort, the average age was 62 and average ΔJSW over 30 months was 0.68 mm. A high correlation between different peptides from individual proteins was observed, indicating our assays correctly measured their target proteins. Peptides representative of clusterin, lumican and lubricin showed statistically significant associations with joint space narrowing after adjustment for age and sex. Partial R^2^ values showed clusterin FMETVAEK and lubricin LVEVNPK peptide biomarkers explains about 2 to 3% of the variability of ΔJSW, similar to that explained by age. A biomarker score combining normalized data for both lubricin and clusterin peptides increased the model R^2^ to 0.079.

**Conclusions:**

Our results suggest that when combined, levels of peptides representative of clusterin and lubricin in plasma are as predictive of OA progression as age. Replication of these findings in other prospective OA cohorts is planned.

**Electronic supplementary material:**

The online version of this article (doi:10.1186/s13075-014-0456-6) contains supplementary material, which is available to authorized users.

## Introduction

Osteoarthritis (OA) is a common, debilitating disease and knee osteoarthritis is a significant public health problem, estimated to cost up to $185 billion per year [1]. Currently, no effective disease-modifying OA drugs are available. The drug development pipeline has been limited by the heterogeneity of OA, the slowly progressive nature of the disease and the fact that there can be long periods of asymptomatic degeneration. Biomarkers that can detect those at risk for disease progression would be beneficial and could minimize the duration and cost of drug discovery [2].

Most biomarkers are proteins and have classically been quantitated with immunoassays. The cost and time needed to develop specific, well-performing antibodies for immunoassays is limiting [3]. Mass spectrometry has emerged as a technology that can measure proteins in routine clinical core laboratories [4] and has the benefit of being able to multiplex analytes and take into account protein variants at the amino acid level [5]. Recent studies have shown mass spectrometry-based assays can have the excellent reproducibility that is necessary for tests in clinical laboratories [6,7].

In a previous study, we used proteomics to identify proteins differentially expressed in OA versus normal synovial fluid [8]. As part of a targeted search strategy for novel OA biomarkers, we developed mass spectrometry assays to measure tryptic peptides representative of several of these proteins, as well as other proteins from previous discovery experiments [9]. These mass spectrometry assays, known as selected reaction monitoring (SRM), use synthetic isotopically labeled peptides for quantitation. We initially developed SRM assays for synovial fluid [8] and subsequently refined them for serum and plasma. The objective of this study was to test a panel of these putative biomarkers in a prospective OA cohort to determine whether peptide concentrations at baseline predict radiographic progression.

## Methods

### Study samples

Synovial fluid and serum samples were collected from participants with full consent and approval under Institutional Review Board (IRB) protocols from Partners Healthcare. The study set consisted of paired synovial fluid and serum samples from 13 patients with late-stage OA taken at the time of joint replacement surgery, and the samples were used to refine our SRM assays.

The progression cohort used for the study was initially described by Mazzuca *et a*l. [10]: 253 subjects were followed for 30 months with serial radiographs and questionnaires. From this cohort, 30% showed radiographic progression over the study period. From the original cohort, 173 subjects were chosen based on the availability of complete radiographic data and BMI <35 to exclude outliers. The plasma used for assays was from the baseline visit. The IRB at Partners Healthcare approved the protocol.

### SRM assays

SRM assays were developed on a TSQ Vantage triple quadrupole mass spectrometer, (Thermo Fisher Scientific, Waltham, MA, USA) as previously described [7,8,11-13]. No depletion of abundant proteins was required. These assays were used to identify potential OA protein biomarkers in paired synovial fluid and serum samples (Table 1).

**Table 1:** **Proteins tested by mass spectrometry in matched synovial fluid and serum samples**

**Protein**	**Protein symbol**	**Peptides, number**	**Synovial fluid**	**Serum**
Aggrecan	ACAN	14	Y	N
Cartilage acidic protein 1	CRTAC1	10	Y	Y
Clusterin	CLU	8	Y	Y
Cartilage oligomeric matrix protein	COMP	15	Y	Y
Dickkopf 1 homolog	DKK1	6	N	N
Fatty acid binding protein 5	FABP5	2	Y	Y
Follistatin	FST	11	N	N
Group XV phospholipase A2	PLA2G15	8	N	N
Hepatocyte growth factor	HGF	1	Y	Y
Hyaluronan and proteoglycan link protein	HAPLN	13	N	N
Insulin-like growth factor-binding complex acid labile subunit	IGFALS	13	Y	Y
Kallistatin	SERPINA4	5	Y	Y
Lumican	LUM	5	Y	Y
Lysosomal phospholipase	ACP2	6	N	N
Matrix metalloproteinase 3	MMP3	10	Y	Y
Pigment epithelium derived factor	PEDF	12	Y	Y
Procollagen C-endopeptidase enhancer 1	PCOLCE	7	Y	Y
Protein S100-A16	S100A16	1	Y	Y
Semenogelin-2	SEMG2	2	Y	Y
Type 1 Collagen (α1, α2)	COL1A	3	Y	Y
Type 2 Collagen (α1)	COL2A1	1	Y	Y
Type 3 Collagen (α1)	COL3A1	1	Y	Y
Xaa-Pro dipeptidase	PEPD	14	N	N

Y, yes; N, no.

To obtain quantitative data from the progression cohort, isotopically labeled heavy peptides (AQUA, Thermo Fisher Scientific) were spiked in each sample. Each sample was digested and analyzed two times in the SRM assay in duplicate for a total of four technical replicates. Coefficients of variation (CVs) ranged from 3 to 15%. The average value of these replicates was used in the analyses. Table 2 lists the 19 peptides representative of 9 proteins in plasma used in these assays.

**Table 2:** **Peptides and proteins assayed by selected reaction monitorin**g

**Protein**	**Peptide sequence**
Afamin	FLVNLVK
Afamin	LPNNVLQEK
Clusterin	TLLSNLEEAK
Clusterin	FMETVAEK
Cartilage oligomeric matrix protein	AVAEPGIQLK
Hepatocyte growth factor	ESWVLTAR
Insulin-like growth factor-binding protein complex acid labile subunit	LAELPADALGPLQR
Insulin-like growth factor-binding protein complex acid labile subunit	TFTPQPPGLER
Kallistatin	LGFTDLFSK
Kallistatin	FFSAQTNR
Lubricin	LVEVNPK
Lubricin	TFFFK
Lubricin	C(-CH_2_-COOH)FESFER
Lubricin	GFGGLTGQIVAALSTAK
Lumican	ISNIPDEYFK
Lumican	FNALQYLR
Pigment epithelium-derived factor	ELLDTVTAPQK
Pigment epithelium-derived factor	TVQAVLTVPK
Pigment epithelium-derived factor	LSYEGEVTK

Thermo Scientific Pinpoint SRM Workflow software was used for targeted protein quantification. This software algorithm is used to predict candidate peptides and for choosing multiple fragment ions for SRM assay design, building an instrument method and a sequence file, and also for automatic peptide identity confirmation and quantitative data processing.

### Knee imaging

All patients underwent standardized radiography including semi-flexed anterior-posterior (AP) views of each knee at the baseline and 30-month follow-up study visit [10]. Both knees were analyzed and the knee with the maximal joint loss during the 30-month follow up was used as the index knee to determine the joint space width for analyses.

### Statistical analysis

We used linear regression to determine the association between each peptide biomarker level at baseline and maximal joint space loss (mm) in the index knee from 0 to 30 months, adjusting for age and sex. A second model adjusting for baseline JSW and body mass index (BMI) was also performed. We obtained *R*^2^ and partial *R*^2^ values for each model to assess the amount of variance explained by the model (*R*^2^) and the relative contribution of the biomarker (partial *R*^2^). Peptides with a marginal or stronger association (*P* <0.2) with joint space loss were advanced for further modeling. A biomarker score was developed using a weighted average of peptides showing preliminary evidence of association (*P* <0.1 or odds ratio (OR) >1.5) in the initial analyses. First, the value for each biomarker was standardized by subtracting the mean and dividing by its standard deviation. Second, the two standardized scores were added to create a biomarker score from the weighted average, with weights equal to the parameters from linear regression. In this way, differing scales for each biomarker did not influence the results. Linear associations between the individual peptide biomarkers were examined using Pearson correlation. As a secondary analysis we examined the association between each peptide biomarker level at baseline and 30-month change in the Western Ontario and McMaster Universities Arthritis Index (WOMAC). All analyses were performed using SAS.

## Results

### Translation of peptide biomarkers from synovial fluid to serum

We initially selected 23 proteins as possible biomarkers (Table 1). These proteins were selected from discovery proteomics experiments comparing OA and normal synovial fluid ([8,9] and data not shown) and lists of previously published OA biomarkers. Multiple peptides generated by tryptic digestion were tested for each of these 23 proteins to refine our assays. Table 1 shows those proteins that were detectible in synovial fluid and/or serum. Peptides representative of six proteins could not be identified in either serum or synovial fluid (lyosomal phospholipase (ACP2), dickkopf 1 (DKK1), follistatin (FST), hyaluronan and proteglycan link protein (HAPLN), group XV phospholipase A2 (PLA2G15), xaa-Pro dipeptidase (PEPD). Peptides representative of one protein, aggrecan (ACAN) were identified in synovial fluid but could not be identified in serum. Most important, the SRM assays were capable of identifying peptides from 16 proteins in both synovial fluid and serum: cartilage acidic protein 1 (CRTAC1), clusterin (CLU), cartilage oligomeric matrix protein (COMP), fatty acid binding protein 5 (FABP5), hepatocyte growth factor (HGF), insulin-like growth factor-binding complex acid labile subunit (IGFALS), kallistatin (SERPINA4), lumican (LUM), matrix metalloproteinase (MMP3), pigment epithelium-derived factor (PEDF), procollagen C-endopeptidase enhancer 1 (PCOLCE), protein S100-A16 (S100A16), semenogelin (SEMG2), type 1 collagen (COL1A), type 2 collagen (COL2A1), and type 3 collagen (COL3A1).

### Measurement of peptide biomarkers in an OA progression cohort

The progression cohort used for this study was a subset of the cohort described by Mazzuca *et al*. [10]. This cohort was predominantly female and obese. Fifty percent had grade-3 OA of the knee at entry into the study [10]. Our subset of subjects had an average age of 62 years and average joint space narrowing over 30 months of 0.68 mm (Table 3). About 83% of the subjects in this cohort had osteophytes and the WOMAC pain score averaged 4.9.

**Table 3:** **Characteristics of 173 subjects at baseline**

**Characteristic**	**Mean (SD) or number (%)**
Age, years	61.8 (9.6)
Body mass index, kg/m^2^	30.5 (5.1)
Sex	
Female	134 (77%)
Male	39 (23%)
Race	
Black	35 (25%)
White	138 (20%)
Years of knee pain	9.0 (9.6)
Years of osteoarthritis diagnosis	5.9 (6.2)
WOMAC pain	4.9 (4.1)
Osteophytes	143 (83%)
Change in joint space width at 30 months	−0.68 (0.64)

WOMAC, Western Ontario and McMaster Universities Arthritis Index.

We selected 19 peptides representative of 9 proteins to test in this cohort. Seven of these proteins were included in the matched synovial fluid/serum study (Table 1). We also included peptides from lubricin, given reports of elevated lubricin expression in the OA joint [14,15], and afamin, which we recently showed to be increased in OA synovial fluid [8]. The proteins and the respective peptides tested in the progression cohort are listed in Table 2. For seven proteins multiple peptides were tested. For ease of identification, when referring to a particular peptide, we use the protein name followed by the first letter of the peptide tested.

All peptides negatively correlated with joint space narrowing, although this correlation did not reach statistical significance. However, peptides for clusterin, lubricin and lumican showed preliminary evidence of association (Table 4). In these models, clusterin(F) and lubricin(L) had the highest *R*^2^ value and significance. In the adjusted models (Table 4, even numbered models), the partial *R*^2^ values of 0.022 for clusterin(F) and 0.033 for lubricin(L) are similar to the partial R^2^ values for age in the models, with significant *P*-values of <0.05. Adding baseline JSW and BMI to the models as additional confounders did not change the relationship between the biomarker and ΔJSW for any peptide. Neither baseline JSW nor BMI were statistically significantly associated with JSW progression in any of the models evaluated (Additional file 1: Table S1).

**Table 4:** **Linear regression models**

**Model number**	**Model** ***R*** ^**2**^	**Parameter**	**Parameter estimate**	**Partial** ***R*** ^**2**^	***P*** **-value**
**1**	0.008	Clusterin(T)	−1.12	0.008	0.25
**2**	0.061	Clusterin(T)	−1.85	0.020	0.06
		Age	−0.01	0.029	0.02
		Sex	0.24	0.023	0.05
**3**	0.008	Clusterin(F)	−0.92	0.008	0.25
**4**	0.063	Clusterin(F)	−1.64	0.022	0.05
		Age	−0.01	0.028	0.03
		Sex	0.26	0.026	0.03
**5**	0.018	Lubricin(L)	−15.03	0.018	0.08
**6**	0.075	Lubricin(L)	−20.96	0.033	0.01
		Age	−0.01	0.030	0.02
		Sex	0.24	0.024	0.04
**7**	0.009	Lubricin(T)	−7.15	0.009	0.22
**8**	0.059	Lubricin(T)	−10.43	0.018	0.07
		Age	−0.01	0.030	0.02
		Sex	0.21	0.019	0.07
**9**	0.011	Lumican(F)	−4.24	0.011	0.17
**10**	0.047	Lumican(F)	−3.15	0.006	0.31
		Age	−0.01	0.018	0.07
		Sex	0.19	0.015	0.10
**11**	0.009	Lumican(I)	−4.14	0.009	0.21
**12**	0.048	Lumican(I)	−3.61	0.007	0.28
		Age	−0.01	0.018	0.08
		Sex	0.20	0.017	0.08
**13**	0.079	Biomarker score^1^	−0.13*	0.038	0.01
		Age	−0.01	0.031	0.02
		Sex	0.28	0.030	0.02

^1^Used standardized beta estimates for Clusterin(F) and Lubricin(L) from models 4 and 6, respectively, to create a weighted sum of the two biomarkers. *One standard deviation of change in biomarker score.

As one biomarker may not be sufficient to predict OA progression, we developed a biomarker score using a weighted average of clusterin(F) and lubricin(L). Using this biomarker score further increased our model *R*^2^ to 0.079 (Table 4, model 13). As a secondary analysis, we looked for correlations between the different peptides for each protein. As expected, peptides that represented the same protein were highly correlated (Table 5, bolded correlations). In contrast, peptides from different proteins had much lower correlations.

**Table 5:** **Pearson correlation matrix for clusterin, lubricin and lumican peptides**

	**Change in joint space width (JSW)**	**Clusterin(F)**	**Clusterin(T)**	**Lubricin(L)**	**Lubricin(T)**	**Lumican(F)**	**Lumican(I)**
**Change in JSW**	1.000	−0.088	−0.088	−0.135	−0.094	−0.105	−0.096
**Clusterin(F)**		1.000	**0.920**	0.514	0.488	0.346	0.356
**Clusterin(T)**			1.000	0.423	0.429	0.401	0.382
**Lubricin(L)**				1.000	**0.958**	0.100	0.105
**Lubricin(T)**					1.000	0.123	0.114
**Lumican(F)**						1.000	**0.858**
**Lumican(I)**							1.000

Values in bold represent strong correlation.

We also looked at how our peptides predicted change in the functional WOMAC scale. The mean change in total WOMAC over 30 months was −1.1 (SD 16.8) (scaled 0 to 100, with 100 being the worst). Pearson correlation coefficients ranged from −0.07 (PEDF(E)) to 0.10 (IGFALS(L)). None of the peptides met the *P* <0.2 criterion for marginal or stronger association. IGFALS(L) displayed the highest *R*^2^ value of 0.0095 (*P* =0.2031).

## Discussion

The development of clinically meaningful medical therapeutics for OA has not progressed for decades. Unlike, for example, rheumatoid arthritis, in which inflammatory markers and autoantibodies portend worse outcomes, OA lacks biomarkers of disease progression. This, combined with the relatively protracted disease course of OA, has made drug development a major challenge. The ability to predict disease progression with a simple blood test would be a major advance.

Here, we have developed plasma-based, mass spectrometry assays to detect peptides representative of proteins we identified as differentially expressed in OA versus normal synovial fluid [8]. In addition, given its importance in OA pathophysiology [16], similar assays to quantify lubricin peptides in plasma were also developed. The high correlation between different peptides for each protein suggests that our peptide assays are representative of the level of the protein they were designed to measure. Levels of our peptides were correlated with maximal change in joint space over 30 months in a well-characterized OA progression cohort. Previously published risk factors in this cohort include age, WOMAC function, contralateral knee OA, ipsilateral patellofemoral OA and baseline JSW [10]. In our adjusted models, the clusterin(F) and lubricin(L) partial *R*^2^ values were similar to age. The finding that our biomarker score was as predictive as age, lends validity to our approach. Although the associations were modest and only clusterin(F) and lubricin(L) achieved statistical significance after adjusting for age and sex, the observation that all peptides negatively correlated with maximal change in joint space suggests our approach may be capturing, in peripheral blood, the ongoing pathophysiology within the OA joint. Supporting this line of reasoning is the biologic relevance of the two proteins (clusterin and lubricin) for which the tryptic peptides in plasma best predicted joint space narrowing in a combined biomarker score.

Clusterin, also known as Apolipoprotein J, is a secreted protein that regulates apoptosis and inflammation. A few studies have observed elevated clusterin in cartilage and synovial fluid in early OA [17,18]. Conversely, decreased clusterin has been observed in rheumatoid arthritis synovial tissues [19]. Clusterin has multiple functions but is best known as a complement regulatory protein where it binds and inactivates the membrane attack complex (MAC) [20]. Interestingly, complement was recently reported to play a role in the pathogenesis of osteoarthritis with mice lacking complement components protected from post-traumatic OA and a demonstration that sub-lytic concentrations of MAC could promote a catabolic phenotype in chondrocytes [21]. The higher levels of clusterin in the OA joint [8] and detected in plasma here may represent activation of a compensatory, but ultimately ineffective, protective pathway. Indeed it has been previously reported that glomerular masangial cells upregulate clusterin expression in response to sub-lytic MAC and in *in vivo* models of glomerular injury [22]. Whether the upregulation of clusterin in OA synovial fluid represents a similar response should be determined.

The other biomarker that performed well in our analyses was tryptic peptide derived from lubricin, also known as proteoglycan 4 and encoded by the *PRG4* gene. This protein is found in synovial fluid, the surface of cartilage, tendons and menisci as well as many other tissues. It is a very large water-soluble glycoprotein with a known role as a joint lubricant [23]. It may also have a role in protecting cartilage surfaces and controlling synovial cell growth [24]. Similar to clusterin, lubricin is upregulated in OA synovial fluid and may also represent activation of a response to local joint injury [14]. Interestingly, despite this upregulation, overexpression of lubricin within the joint in mouse models of OA is protective, suggesting the endogenous response in OA is insufficient [15]. Thus, lubricin is both an attractive biomarker and potential therapy for OA.

Investigations into biomarkers of OA have been underway for more than 20 years yet none are in clinical practice [25]. Urinary C-terminal telopeptide of collagen type II (uCTX-II) and COMP have been studied for many years as burden of disease and prognostic biomarkers [26-28]. A recent community-based cohort study showed higher COMP predicting incident OA over the next 6 years [28]. Lately, interest in this area has increased with the identification of new candidate biomarkers for progression. More recent additions include the alarmins S100A8/S100A9 [29], showing higher levels in those who progressed over 2 years, adipokines, such as leptin and resistin, which predict progressive and incident OA respectively [30], and TNF-stimulated gene 6 (TSG-6), which predicts radiographic progression over 3 years [31]. Some studies suggest that panels of biomarkers may better predict outcomes and that there is additive value in combining multiple markers [32,33].

This study introduces an approach for protein biomarker development to the OA field, which allows for rapid assay development, SRM mass spectrometry, to identify tryptic peptides representative of target proteins. Mass spectrometry is being increasingly used in clinical laboratories where it has been shown to be simple, robust, precise and less costly than traditional immunoassays [3,5,34,35]. Moreover, the ability to multiplex analytes in very small volumes of samples is another strength of this technology. The assays used for this publication required only 25 μL of sample yet were able to identify many different peptides. Using freely available peptide information from online repositories [36], peptides can be selected and rapidly tested in SRM assays. If detailed biology is available about post-translational modifications of proteins, this can be incorporated into the design of the assays as well [37].

A strength of this work is the large number of plasma samples analyzed from the progression cohort. Furthermore, the cohort was followed prospectively with standardized radiographs and with our SRM assays we were able to generate quantitative and reproducible data for our target peptides. Our study has important limitations. We performed an index knee analysis, whereby only the knee with the greatest change in JSW was considered. The contralateral knee, which may or may not have had progressive OA, was not taken into account. In addition, our analysis was not powered to predict change in WOMAC and did not incorporate relative differences in the change in joint space in the medial versus the lateral compartments or osteophyte progression, as these data were not available. The variability of our peptides among our samples was limited, restricting our ability to detect changes that might be associated with joint space narrowing. In addition, this cohort already had OA, with a majority having at least grade-3 radiographic disease. As some of our proteins were selected based on differences in the synovial fluid from subjects with normal and late OA, the advanced disease in our cohort may have made it challenging to detect major associations between these biomarkers and change in joint space. Likewise, few differences were noted in the synovial fluid proteome of knee OA patients with early and late disease [8,9], suggesting the disease process is entrenched by the time symptoms appear. Thus, measurement of our biomarkers in cohorts of patients with pre-radiographic disease is planned to see if these peptides predict OA progression. A further weakness is that our biomarkers were only measured at the baseline time point. Thus, it is unknown whether these biomarkers change over time, and if this change may be more predictive of OA progression than a baseline measurement. Finally, our data focused on knee OA, so we cannot make any generalizations to other forms of OA based on our data.

## Conclusions

In conclusion, targeted mass spectrometry assays provide a rapid means to measure novel biomarkers in human samples. Our results suggest that when combined, clusterin and lubricin peptide levels in plasma are as predictive of OA progression as age. Replication of these findings in prospective progression and incidence OA cohorts is planned.

## Additional file

Additional file 1: Table S1. Linear regression models adjusting for age, sex, body mass index (BMI) and baseline joint space width (JSW).

## Abbreviations

ACAN: aggrecan
ACP2: lyosomal phospholipase
BMI: body mass index
CLU: clusterin
COL1A: type 1 collagen
COL2A1: type 2 collagen
COL3A1: type 3 collagen
COMP: cartilage oligomeric matrix protein
CRTAC1: cartilage acidic protein 1
DKK1: dickkopf 1 homolog
FABP5: fatty acid binding protein 5
FST: follistatin
HAPLN: hyaluronan and proteglycan link protein
HGF: hepatocyte growth factor
IGFALS: insulin-like growth factor-binding complex acid labile subunit
IRB: Institutional Review Board
JSW: joint space width
LUM: lumican
MAC: membrane attack complex
MMP: matrix metalloproteinase
OA: osteoarthritis
PCOLCE: procollagen C-endopeptidase enhancer 1
PEDF: pigment epithelium-derived factor
PEPD: xaa-pro dipeptidase
PLA2G15: group XV phospholipase A2
S100A16: protein S100-A16
SEMG: semenogelin
SERPINA4: kallistatin
SRM: selected reaction monitoring
TNF: tumor necrosis factor
uCTX-II: urinary C-terminal telopeptide of collagen type II
WOMAC: Western Ontario and McMaster Universities Arthritis Index
ΔJSW: change in joint space width
