# Supplementary material for: Mass spectrometry assays of plasma biomarkers to predict radiographic progression of knee osteoarthritis
Source: Arthritis Res Ther. 2014 Oct 7;16(5):456. doi: 10.1186/s13075-014-0456-6 (PMC4207325; doi:10.1186/s13075-014-0456-6)
Supplement: Additional file 1: Table S1. — Linear regression models adjusting for age, sex, body mass index (BMI) and baseline joint space width (JSW). [file 13075_2014_456_MOESM1_ESM.docx]

**Table S1. Linear regression models adjusting for age, sex, BMI, and baseline JSW**

| **Model #** | **Model R^2^** | **Parameter** | **Parameter Estimate** | **Partial R^2^** | **p-value** |
| --- | --- | --- | --- | --- | --- |
| 1 | 0.071 | Clusterin_T | -1.87 | 0.020 | 0.06 |
|  |  | Age | -0.01 | 0.026 | 0.03 |
|  |  | Sex | 0.24 | 0.023 | 0.04 |
|  |  | BMI | -0.00 | 0.001 | 0.71 |
|  |  | BL JSW | 0.05 | 0.009 | 0.20 |
| 2 | 0.073 | Clusterin_F | -1.65 | 0.022 | 0.05 |
|  |  | Age | -0.01 | 0.025 | 0.04 |
|  |  | Sex | 0.26 | 0.026 | 0.03 |
|  |  | BMI | -0.00 | 0.000 | 0.81 |
|  |  | BL JSW | 0.05 | 0.009 | 0.20 |
| 3 | 0.084 | PRG4_L | -22.39 | 0.033 | 0.02 |
|  |  | Age | -0.01 | 0.025 | 0.04 |
|  |  | Sex | 0.24 | 0.023 | 0.04 |
|  |  | BMI | 0.00 | 0.001 | 0.69 |
|  |  | BL JSW | 0.05 | 0.009 | 0.21 |
| 4 | 0.068 | PRG4_T | -10.82 | 0.017 | 0.08 |
|  |  | Age | -0.01 | 0.025 | 0.03 |
|  |  | Sex | 0.21 | 0.018 | 0.07 |
|  |  | BMI | 0.00 | 0.000 | 0.88 |
|  |  | BL JSW | 0.05 | 0.008 | 0.22 |
| 5 | 0.057 | Lumican_F | -3.21 | 0.006 | 0.31 |
|  |  | Age | -0.01 | 0.017 | 0.09 |
|  |  | Sex | 0.19 | 0.016 | 0.10 |
|  |  | BMI | -0.00 | 0.001 | 0.65 |
|  |  | BL JSW | 0.05 | 0.009 | 0.22 |
| 6 | 0.058 | Lumican_I | -3.77 | 0.007 | 0.27 |
|  |  | Age | -0.01 | 0.016 | 0.09 |
|  |  | Sex | 0.21 | 0.018 | 0.08 |
|  |  | BMI | -0.00 | 0.001 | 0.65 |
|  |  | BL JSW | 0.05 | 0.009 | 0.21 |
| 7 | 0.089 | Biom_Score1* | -0.139 | 0.038 | 0.01 |
|  |  | Age | -0.01 | 0.026 | 0.03 |
|  |  | Sex | 0.27 | 0.029 | 0.02 |
|  |  | BMI | 0.00 | 0.001 | 0.74 |
|  |  | BL JSW | 0.05 | 0.009 | 0.19 |

* Used standardized beta estimates for Clusterin(F) and Lubricin(L) from models 4 and 6 respectively to create a weighted sum of the two biomarkers

† 1 standard deviation of change in biomarker score
